# Development of a Pediatric Relative Bioavailability/Bioequivalence Database and Identification of Putative Risk Factors Associated With Evaluation of Pediatric Oral Products

**DOI:** 10.1208/s12248-021-00592-y

**Published:** 2021-04-21

**Authors:** Gopal Pawar, Fang Wu, Liang Zhao, Lanyan Fang, Gilbert J. Burckart, Kairui Feng, Youssef M. Mousa, Franci Naumann, Hannah K. Batchelor

**Affiliations:** 1 School of Pharmacy, Institute of Clinical Sciences, University of Birmingham, Edgbaston, B15 2TT UK; 2 Division of Quantitative Methods and Modelling, Office of Research and Standard, Office of Generic Drug Products, Center for Drug Evaluation and Research, United States Food and Drug Administration, Silver Spring, Maryland 20993 USA; 3 Office of Clinical Pharmacology, Office of Translational Science, Center for Drug Evaluation and Research, United States Food and Drug Administration, Silver Spring, Maryland 20993 USA; 4 Strathclyde Institute of Pharmacy and Biomedical Sciences, University of Strathclyde, 161 Cathedral Street, Glasgow, G4 0RE UK

**Keywords:** bioequivalence, oral product, pediatric, relative bioavailability, risk factors

## Abstract

Generally, bioequivalence (BE) studies of drug products for pediatric patients are conducted in adults due to ethical reasons. Given the lack of direct BE assessment in pediatric populations, the aim of this work is to develop a database of BE and relative bioavailability (relative BA) studies conducted in pediatric populations and to enable the identification of risk factors associated with certain drug substances or products that may lead to failed BE or different pharmacokinetic (PK) parameters in relative BA studies in pediatrics. A literature search from 1965 to 2020 was conducted in PubMed, Cochrane Library, and Google Scholar to identify BE studies conducted in pediatric populations and relative BA studies conducted in pediatric populations. Overall, 79 studies covering 37 active pharmaceutical ingredients (APIs) were included in the database: 4 bioequivalence studies with data that passed BE evaluations; 2 studies showed bioinequivalence results; 34 relative BA studies showing comparable PK parameters, and 39 relative BA studies showing differences in PK parameters between test and reference products. Based on the above studies, common putative risk factors associated with differences in relative bioavailability (DRBA) in pediatric populations include age-related absorption effects, high inter-individual variability, and poor study design. A database containing 79 clinical studies on BE or relative BA in pediatrics has been developed. Putative risk factors associated with DRBA in pediatric populations are summarized.

**Supplementary Information:**

The online version contains supplementary material available at 10.1208/s12248-021-00592-y.

## INTRODUCTION

To ensure therapeutic equivalency, the rate and extent of drug absorption should not be significantly different between generic products and their corresponding reference products when administered at the same molar dose of the therapeutic ingredient under similar experimental conditions in either a single dose or multiple doses (1, 2). BE studies form an integral part in generic and new drug products approval process. For assessing BE, PK endpoints such as peak plasma concentration (Cmax) and area under the plasma concentration time curve (AUC), which reflect the rate and extent of drug absorption, are used. According to the U.S. Food and Drug Administration (U.S. FDA), the test and reference products are considered BE when the 90% confidence interval of the geometric mean ratios of Cmax, AUC_0–t_, and AUC_0–∞_ between the test and reference formulations are within the BE acceptance region of 80–125% (3). Because the purpose of a BE study is to draw a generalizable conclusion about the test and reference products, BE studies are most often conducted in healthy adult populations. BE studies are designed to compare the test and reference products with the same dosage form in the most sensitive, accurate, and reproducible way. From this evidence, regulators then conclude that the test and reference products can be substituted for all patient populations described in the product label.

Due to the need for age-appropriate medicines with flexible dosing options, pediatric formulations can differ from the product used in adults (e.g., use of a suspension rather than a tablet); thus, a change in relative BA may be possible. Indeed, the pediatric formulation is designed to achieve the target systemic exposure in the population of interest thus a change in PK exposure may be attributable to formulation design. The purpose of relative BA studies, as distinct from BE studies, is to compare the rate and extent of drug absorption between two pharmaceutical dosage forms (e.g., a novel pediatric formulation) and a reference product (e.g., adult formulation); they are usually conducted in healthy adults (4). Moreover, there is no formal statistical assessment associated with relative BA studies to demonstrate whether they “pass” or “fail” yet sometimes BE acceptance limits are applied to these studies (5). Because relative BA studies are often designed to be descriptive and intended to inform instructions for product use, it is more common to find a relative BA study conducted in a pediatric population than to find a BE study conducted in a pediatric population.

Drug absorption differs in pediatric populations compared to adults due to differences in gastrointestinal (GI) physiology and anatomy (6, 7). This has led to concerns about comparing pediatric drug product formulations in healthy adults to inform treatment in pediatric populations. Healthy adults are often used as a surrogate population to test the BE of generic and reference pediatric products and to test relative BA for novel pediatric formulations via a two-way crossover design. Thus, the results from adult population studies (i.e., BE and relative BA studies) are presumed to be the same as in the pediatric population. However, there is a need to identify drug substance or drug products under certain circumstances at risk of discrepancy with the above assumption, so further control strategy/justification can be applied to these products.

When a relative BA study is conducted in adults, the magnitude of difference between the two formulations is often directly applied to the pediatric population to decide the appropriate dose to administer. This was the case with the lamivudine ARROW trial (8), which compared split scored-tablets and solutions of lamivudine in children and showed that the exposure was approximately 55% higher with the tablet compared to the liquid in children yet the two formulations were equivalent in an adult population (9). The causative agent in this case was presumed to be the sorbitol content of the oral liquid, which influences the GI transit time (10). Thus, an understanding of pediatric anatomy and physiology is required to better predict the impact of drug product dissolution and drug substance solubility on drug absorption. For instance, several reports showed changes in the Biopharmaceutics Classification System (BCS) class for the same drug substance in pediatric compared to adult populations (11–15). A number of risk factors linked to bioinequivalence have been identified from the literature, some examples include: physiological factors (absorption-distribution-metabolism-excretion, ADME effects) (16), formulation effects (10), disease progression and other disease-related effects (17), and poor study design (18).

Despite the notion that BE established in adults is generally extrapolatable to pediatric populations, there is still a need to identify putative risk factors associated with this concept. This study intended to collect available data from literature that reported bioinequivalence or disparities in PK profiles in BE and relative BA studies conducted in pediatric populations to improve the understanding and identification of any common risk factors that relate to the difference in PK observed.

## METHODS

We systematically searched the literature in PubMed (19), Cochrane Library (20), and Google Scholar (21) for potentially relevant studies that assessed relative BA and BE studies in a pediatric population. To collate the largest possible datasets, different search keywords were identified from the literature such as: “bioequivalence” (22); “relative bioavailability” (16); “failed bioequivalence” (23); “lack of bioequivalence” (24); “bioinequivalence” (25), and “non-bioequivalent” (26). In this paper, we used the term “bioinequivalence” to describe cases where the BE criteria were not met and to encompass lack of BE, failed BE, or non-bioequivalent. The combination of search keywords listed in Table I were used to capture data from pediatric populations (aged from 0 to 17 years).

**Table I:** Combination of Search Keywords Used To Identify Relevant Literature. Note for Google Scholar, We Omitted “OR” and “AND” With the Keywords

To identify relevant studies	“Bioequivalence” OR “Relative Bioavailability” OR “Non-Bioequivalent” OR “Failed Bioequivalence” OR “Lack of Bioequivalence” OR “Bioinequivalence”
	AND
To limit to a pediatric population	“Infant” OR “Child” OR “Children” OR “Adolescent” OR “Pediatric”
	AND
To limit to orally administered products	“Oral drug”

Any duplicate studies identified in more than one database were excluded from the list with the help of EndNote and manual review.

The inclusion and exclusion criteria used to identify relevant high-quality studies are listed in Table II. Two independent reviewers applied these criteria to the identified literature to minimize bias.

**Table II:** Inclusion and Exclusion Criteria Used To Identify the Studies To Be Included in Further Analysis

Inclusion criteria	• Studies conducted on U.S. FDA or European Medicines Agency (EMA) approved drugs for oral administration only. • Studies must include data from pediatric populations. • The studies must provide information on study design (e.g., randomized controlled, crossover design, and parallel design), subjects information (age, weight, height, sex, origin, inclusion, or exclusion criteria), sample size, dose of the drugs (single or multiple), washout period, study conditions (fasting or fed state), and clinical trials registration ID. • BE studies must report the statistical analysis containing the 90% CIs (80–125%) or geometric mean ratios (0.8–1.25) for both the test and reference medicines for the PK endpoints AUC and Cmax. Studies should also state whether they met the BE criteria according to U.S. FDA or EMA guidelines. • For relative BA studies, PK endpoints such as AUC and Cmax data are required for tested and reference products.
Exclusion criteria	• Studies on drugs not administered orally. • Studies reporting difference in relative bioavailability (DRBA) due to the presence of food or drinks or herb-drug interactions or drug-drug interactions.

A standardized data collection tool was constructed using Microsoft Excel to capture the extracted information under different categories as provided in Table III.

**Table III:** Details of Information Extracted From Each Study Identified Within the Literature Search

Study details	Study title; URL link; study design (i.e., randomized controlled, parallel, or cross-over); sample size; dose administered; single or multiple dose
Study population	Age; healthy/diseased state; number of participants
Test and reference products	Dosage form and strength of the test and reference products (i.e., tablet and capsule); pre-dosing manipulations (e.g., halving or quartering or crushing the tablets before administration)
Administration details	Fasted/fed state; volume of water consumed (ml)
Study results	90% CIs (80–125%) or geometric mean ratios (0.8–1.25) for both the test and reference medicines for the PK endpoints AUC and Cmax; statistics
Interpretations	Authors interpretation of data on product equivalence (Yes/No)
Risk factors for DRBA	Original authors’ reasons for DRBA stated in the paper
Clinical impact of DRBA	Authors interpretation of data on clinical equivalence (Yes/No)
Miscellaneous details	Clinical trials registration ID; remarks; reference

In this paper, we use the term, difference in relative bioavailability (DRBA), to refer to either failed BE studies or studies that demonstrated a difference in relative bioavailability. In this paper, we extracted the reasons that the original authors provided for the change in exposure noted within the studies. This means that the ADME-related differences were those reported in the original studies and it may well be an interplay between the formulation and the absorption This could also include links to secondary references where authors interpreted their data in the context of wider literature.

The physicochemical drug properties, including molecular weight, Log P, and water solubility, as well as oral BA (%), were extracted from the Drug Bank database (27). Narrow therapeutic index (NTI) data for the drugs where “small differences in dose or blood concentration may lead to dose and blood concentration dependent, serious therapeutic failures or adverse drug reactions that are life-threatening or result in persistent or significant disability or incapacity” (28) were identified using information within product-specific guidances (PSG) where the U.S. FDA identified drugs as NTI (29). The BCS classification for those drugs included was reported from the NICHD-FDA report (30) and from specific publications relating to the development of provisional pediatric BCS systems (6, 14, 15, 31). Both adult and pediatric BCS classifications are reported to enable comparison within these populations and to identify those drugs where the BCS classification changes between adult and pediatric populations.

## RESULTS

A total of 79 clinical studies containing data from pediatric populations were identified using the search terms listed above and applying the inclusion and exclusion criteria (Supplementary Material 1, database for relative BA and BE studies in pediatrics). Subsequently, a total of 41 studies with DRBA results between test and reference products remained for further analysis (Supplementary Material 2, risk factor summary). The 41 studies included 2 BE studies and 39 relative BA studies that showed disparities in PK properties when test and reference produces were compared.

These 41 (2 BE and 39 relative BA) studies were interrogated to identify the reported putative risk factors that could be responsible for the failure of BE in pediatrics. A summary of these risk factors is shown in Table IV.

**Table IV:** Summary of Stratification of Author’s Reported Reasons for Failed Pediatric BE and Relative BA Studies Identified Within This Review. Note That Multiple Reasons May Have Been Listed for a Single Study

	Putative risk factors	Number of studies identified	References
Physiological factors (ADME effect)	Age-related absorption effects (e.g., GI motility, GI fluid volume or composition, and GI transit time)	28	(8, 26, 32–57)
	Age-related distribution effects (e.g., protein binding)	2	(40, 47)
	Age-related metabolism or clearance effects	15	(32–34, 36, 42, 43, 47–50, 58–62)
Drug substance or formulation effects	Drug substance effect (e.g., alternative salt or polymorphic form of drug substance)	5	(34, 35, 39, 40, 51)
	Drug product/formulation effects	12	(32, 34–41, 43, 45, 63)
Disease	Age-related disease progression and other disease-related effects	4	(26, 53, 63, 64)
Population characteristics	High inter- and/or intra-individual variabilities	18	(33, 36–39, 41, 45–48, 50, 54, 55, 58, 61, 63, 65, 66)
Study design	Non-equivalent dose effects	2	(39, 67)
	Accuracy of administered dose	2	(60, 68)
	Poor study design including small sample size	11	(35, 43, 44, 49, 51–53, 59, 62, 66, 67)

Further details and examples of studies are described in the text below.

### Physiological Factors (ADME Effect)

#### Age-Related Absorption Effects

As listed in Table IV, 28 studies reported DRBA attributed to age-related absorption (differences in absorption between pediatric and adult populations). Fifteen out of 28 studies reported multiple absorption-related risk factors (8, 32–45), including alterations in GI transit time or motility (8, 33, 36, 38, 40, 44), changes in GI fluid composition (37, 40), and different levels of saturation for intestinal transporters/metabolism (8, 42). Moreover, 11 of the studies from the list of 28 reported factors are also related to the formulations used (32, 34–41, 43, 45), and 5 also related to the drug substance (8, 33, 38, 40, 44). It should be noted that a BE study conducted as a crossover study would cancel out the age-related effects on clearance and highlight those effects that are related to formulation differences; however, due to the limited crossover BE studies conducted in pediatric populations, we summarized all the risk factors related to applying clinical study results from adults to pediatric populations.

##### GI Transit

Altered GI transit time in children as compared to adults was listed as a risk factor for DRBA. For example, an emtricitabine capsule formulation provided 20% higher plasma exposure compared to the solution formulation in children reportedly due to the fact that the GI transit time of the solution was shorter than that of the capsule formulation (44).

The rapid GI transit in children has been implicated as a risk factor for DRBA for drugs such as cyclosporine A (33, 36, 38). This risk factor is associated with formulation changes that alter the rate of drug release (33, 36, 38, 46). In previous studies conducted in adults, factors including bile flow, GI motility, bowel length, and food intake and composition were found to affect the absorption of certain cyclosporine formulations (38) and phenytoin (40). There is an interplay between formulation effects and GI transit, and if GI transit time limits drug absorption, differences in formulation may have impact on the absorption. Thus, the implications of these effects in adult studies may increase in pediatric populations (40).

In neonates and infants below the age of 6–8 months, gastric emptying was found to be slower due to immaturity of the neuroregulation of gastric motility (69). Evidence on differences in GI transit between adults and children is conflicting and there is very limited detailed information on gastric emptying and small intestinal transit times in pediatric populations (70, 71). There is evidence that shorter transit times of solutions compared to solid dosage forms in adults were observed from one study comparing solutions, small pellets, and tablets in adults (72) and from a second study comparing the pharmacokinetics of alendronate solution and tablets (73). Thus, a change in formulation from solid to liquid may be associated with shorter transit time in pediatric populations; this is likely to be exacerbated for monolithic controlled release tablets as their relative size may delay passage through the pyloric sphincter, as observed with a quicker onset of action for multi-particulate formulations compared to single unit tablets (74, 75). However, it should be noted that there is currently no evidence on the relative gastric emptying or intestinal transit time of liquid and solid oral dosage forms in pediatric populations.

##### Saturation of GI Transporters

The authors reported that oral administration of highly soluble drugs may lead to very high local concentrations of active components in children resulting in saturation of GI membrane influx or efflux transporters leading to altered BA (8). For liquid dosage forms, the drug is already homogenously dissolved or dispersed within the solvent yet dissolution from solid oral dosage forms can lead to regions of highly concentrated drug in solution. The impact of high local concentrations saturating GI membrane transporters was identified as a risk factor in the higher exposure of lamivudine tablets compared to an oral solution in children (8). Saturation of GI membrane transporters was also identified for a BCS class 3/1 drug sulfadoxine (76) as a risk factor in a study where there was a 32% reduction in the relative BA of sulfadoxine (in combination with pyrimethamine) when the dose was doubled in infants with malaria (42). Previously, the concept of saturation of efflux transporters has been highlighted as a theory yet not considered to be clinically relevant (77, 78); thus, further research is required to better understand the true likelihood of saturation of transporters in pediatric populations leading to clinically significant changes.

#### Age-Related Distribution Effects

Two studies were identified with DRBA attributed to differences in the volume of distribution (40, 47): one specifically identified protein binding (40) and another study identified intracellular drug transport as risk factor for DRBA (47).

The volume of distribution of drugs has been reported to change with age. In younger children, higher doses of hydrophilic drugs are required because of the higher percentage of water content in their body; however, lower doses of hydrophilic drugs are required in older children due to the increased proportion of body fat (79). Neonates have lower plasma protein bindings which may increase the fraction unbound for certain highly protein bound drugs and lead to higher plasma exposure (80).

#### Age-Related Metabolism or Clearance Effects

Fifteen studies were identified with DRBA due to changes in metabolism or clearance (32–34, 36, 42, 43, 47–50, 58–62). Differences in enzyme expression and activity can result in altered metabolism of drug substance, which may result in altered production or accumulation of the metabolites. A classic example is gray baby syndrome where the immaturity of hepatic UDP-glucuronyl transferase enzymes in babies leads to accumulation of chloramphenicol metabolites and subsequent toxicity (81).

Variability in genetic polymorphism associated with certain metabolic pathways may increase the likelihood of DRBA (82). This was implicated as a risk factor in a study on lopinavir/ritonavir which are substrates of the multi-drug resistance gene (MDR-1) and organic anion-transporting-polypeptide (OATP) drug transporters as well as some CYP450-based metabolism in the GI tract (47).

Risks for DRBA were also reported (in 1986) for alternative phenytoin (generic tablets and capsules) in epileptic children where dose-related saturation of hepatic metabolizing enzymes or binding proteins could lead to higher plasma concentrations as a result of the small change in dose/exposure resulting in toxicity (49).

Since several other studies conducted in adults have highlighted issues changing phenytoin brand (83) for reasons including a change in excipients (from calcium sulfate to lactose) (84); differences in the physicochemical properties of excipients (85); differences in uniformity of content (86); stability issues (86), and ethnic differences in metabolism (87). Current advice in the UK is to remain on the same brand of phenytoin where possible (88). In addition, as FDA has concluded that phenytoin is an NTI drug (within the product-specific guidance), thus the *in vivo* BE study should be a fully replicated crossover design in order to scale bioequivalence limits to the variability of the reference product and meet the unscaled average BE criteria and compare test and reference product within-subject variability (89).

### Drug Substance and Formulations Effects

#### Drug Substance Effects

Five of the identified studies reported drug substance properties (e.g., salt form, intrinsic solubility, and crystal form) as factors associated with DRBA (34, 35, 39, 40, 51). Here, “drug substance” refers to the active ingredient and includes properties related to particle size, salt form, and intrinsic solubility. For drugs such as rifampicin and tacrolimus, poor solubility has been highlighted as one of the confounding reasons for DRBA (35, 51).

DRBA between hydrocortisone suspension (Cortef: 10 mg/ml, suspending agent-xanthan gum) and a tablet was observed in 19 children; as a result, the liquid formulation was recalled from the market (39). The liquid formulation in this study contained hydrocortisone cypionate, an ester form of hydrocortisone which is different from the free hydrocortisone used in previous suspension formulation used in adult study (90). In adult study, the suspension formulation of free hydrocortisone showed higher absorption compared to the tablets and a shorter half-life than hydrocortisone tablets; they are not BE to each other either.

The crystal form plays an important role in the absorption of pharmaceutical active ingredient. The manufacturing process of rifampicin (RMP) could result in the formation of either polymorphic or amorphous state of RMP with eightfold difference in water solubility between the two states (91). Consequently, RMP exposure was found to be 76% lower among children who received the R-Cin® suspension compared to Eremfat®. The study highlighted that the differences in BA between the two suspension formulations were due to the mixture of polymorphic forms of RMP in R-Cin® that was not favorable for absorption (51). The solubility of any material is dependent upon the volume and composition of the solvent; there are known differences in the intestinal fluids between adults and children thus the relative difference in the formulation may not be directly transferable.

The particle size of sodium phenytoin can affect its oral BA; three separate studies have demonstrated that smaller particles dissolve more rapidly and have increased BA compared to larger particles (34, 40, 92).

The intrinsic poor solubility of certain drugs can increase risks of non-linear PK due to incomplete dissolution within the GI tract (35, 49, 52, 63), especially for children whose GI volume is less compared to adults (93).

#### Drug Product/Formulations Effects

Drug product effects may change the exposure to a drug substance due to changes in dosage form (e.g., tablet versus capsule or liquid oral formulation), drug release profiles (e.g., immediate or extended release or complex release formulation), and formulation constituents (e.g., excipient effect). Drug product effects are typically reflected in relative BA studies.

From the relative BA studies identified, 12 studies showed drug product effects on BA (32, 34–41, 43, 45, 63). The magnitude of drug product effects may be amplified in pediatric populations where differences in absorption relate to factors such as GI motility where small formulation changes can result in large PK differences as shown with controlled release formulations (33, 38, 40, 44).

Neoral (microemulsion of cyclosporin A) was rapidly absorbed in children producing a high peak level within 1 to 2 h of dosing. This observation was due to the increased effective surface area achieved by the microemulsion compared to a more conventional formulation (33, 36–38, 45).

Three studies reported DRBA due to excipient effects (32, 34, 63). For extemporaneously prepared 6-mercaptopurine (6-MP) liquid formulations, differences in formulation constituents (excipients) and viscosity, and potentially 6-MP could have contributed to the observed variability in systemic drug availability from liquid formulations (63). Lactose, which is an excipient widely used as a filler or filler-binder in the manufacture of tablet and capsules, could aid in moistening of the phenytoin particles in tablet formulation and this enhanced the dissolution rate followed by increase in absorption and plasma exposure (34).

### Age-Related Disease Progression and Other Disease-Related Effects

FDA’s Guidance for Industry: Providing Clinical Evidence of Effectiveness for Human Drug and Biological Products recommends that the efficacy data from adult population can be extrapolated to pediatric population when the course of the disease and the effects are sufficiently similar (94). In our analysis, 4 studies attributed the DRBA reported to differences in disease progression between adults and children (26, 53, 63, 64). These diseases include HIV; congenital hypothyroidism; malaria; epilepsy; attention deficit hyperactivity disorder (ADHD), and organ transplant. For example, in HIV-infected children, co-administration of antibiotics with anti-HIV drugs have been reported to (i) decrease the absorptive surface area of the intestine (95), (ii) alter gut flora (96), (iii) cause local GI inflammation (97), (iv) lead to fat malabsorption (98), and (v) cause diarrhea (99). All of these effects were shown to alter the BA of the anti-HIV zidovudine in children (100). In another example, Synthroid and one of its generic L-T(4) versions were observed to have a different pharmacodynamic response in children (*n* = 36) suffering from congenital hypothyroidism (CH) due to diminished thyroid reserve (26).

### Population Characteristics

A total of 18 studies linked DRBA to population effects from participants in the study. These population effects include: high inter-individual variability within the study population (33, 36–39, 41, 45–48, 54, 55, 58, 61, 63, 65, 66), high intra-individual variability (45, 50, 63), or genetic polymorphism associated with metabolism of drugs (47, 58, 65). The consequences of these population effects may increase in the developing pediatric population. For example, pediatric liver transplant recipients are known to show poor and variable absorption of cyclosporin partly because of the limited absorptive surface area associated with small bowel lengths (36, 101), surgical bowel reconstruction, and also due to higher rates of metabolism of cyclosporine (36, 55).

### Study Design

Dosing issues and poor study design were listed as a risk factor for DRBA in 15 studies, as detailed in Table IV.

#### Non-equivalent Dose and Accuracy of Administered Dose

Non-equivalent dose may play a role in the DRBA of the tested medicines such as hydrocortisone and levothyroxine (39, 67). In addition, extemporaneous preparation of products such as hydrocortisone and nevirapine may make dose accuracy within the test questionable (60, 68).

#### Poor Study Design Including Small Sample Size

Failure to meet BE criteria may occur as a result of underpowered studies (102). Eleven studies reported DRBA due to an underpowered study design (35, 43, 44, 49, 51–53, 59, 62, 66, 67).

Small sample size was a factor behind the DRBA reported in 7 of the original 11 studies (43, 44, 51, 53, 59, 66, 67). One of the studies already highlighted recruited small sample sizes (*n* = 2–10) due to the nature of study population, for example, patients with organ transplant (59). Risk factors of poor study design from the studies already identified include: non-randomized study design (44, 62, 67, 103); parallel study arms (35); retrospective study design (104); methodology used in statistical results (e.g., partial AUC calculations were not considered for complex release methylphenidate formulations) (104), or baseline errors for endogenous molecules (e.g., levothyroxine (105)).

### Further Analysis on BCS Classification of Drugs Showing DRBA Results

The BCS classification of the drugs included in the studies were reported as those based on adult data and those relevant to the pediatric data as described in “METHODS.” Table V summarizes the BCS classification and NTI of each drug where DRBA was reported.

**Table V:** BCS Classification of Drugs According to Adult and Provisional Pediatric BCS System

Drugs showing DRBA	Adult BCS class	Pediatric BCS class	Type of study	Representative reference(s)
**Carbamazepine**	2	2	Relative BA	(48)
**Cyclosporine**	2	2	Relative BA	(57)
Efavirenz	2	2	Relative BA	(43)
Emtricitabine	1	1^*^	Relative BA	(44)
Hydrocortisone	1	3/4^#^	Relative BA	(39)
Indinavir	2/4	2/4^*^	Relative BA	(41)
Lamivudine	3	3	Relative BA	(8, 64)
**Levothyroxine**	1	1	Relative BA BE	(67) (26)
Lopinavir	2	2	Relative BA	(66, 106)
6-Mercaptopurine	4	2^#^	Relative BA	(63)
Nevirapine	2	2	Relative BA	(58)
**Phenytoin**	2	2	Relative BA	(34, 49)
Pyrimethamine	4	2^#^	Relative BA	(42)
Ritonavir	2	2	Relative BA	(66, 106)
Rifampicin	2	2	Relative BA	(51)
Stavudine	3	3	Relative BA	(64)
Sulfadoxine	3/1	3/1^*^	Relative BA	(42)
**Tacrolimus**	2	2^*^	Relative BA BE	(52, 59) (103)
**Vitamin E**	Not classified	Not classified	Relative BA	(107)

^*^Drug BCS class in pediatric is assumed to be same as that of adult BCS class where no provisional pediatric BCS class has been assigned for these drugs

^#^4 Drugs exhibit BCS class shift (2 favorable (BCS 4 to 2) and 2 non-favorable shift (BCS 1 to 3 and BCS 3 to 4)). Drugs name in bold are NTI drugs otherwise non-NTI class

BCS 2 drugs were the most commonly reported with DRBA, perhaps unsurprisingly as poor solubility is associated with greater variability in plasma exposure (108, 109). The proportion of BCS 2 drugs (55.5%) listed here was lower to the proportion of BCS 2 drugs mentioned in a previous study (based on AUC criteria in healthy, young volunteers) where 63% of drugs were BCS class 2 DRBA products (110).

### Further Analysis on NTI Classification of Drugs Showing DRBA Results

In this study, the NTI drugs made up 28% of those where DRBA was reported. The proportion of NTI drugs within this study appears to be lower than the proportion previously reported in a similar study (based on a small subset of products from 2005 to 2008) (28% in this study compared to 56% in the previous study) (22) (111). However, it should be noted that the previous study used a much smaller sample size (*n* = 9 DRBA studies) where data mining was conducted for 3 years and only 9 DRBA studies were identified out of 79 articles in their analysis. The high proportion of NTI drugs where DRBA was reported in the current study is unsurprising indicating that NTI is a risk factor and the challenges in demonstrating bioequivalence for NTI products have already been highlighted (112).

The data shows that the proportion of those drugs showing DRBA that were NTI drugs is highest in the BCS 2 and then followed by 3 and 1 (highly soluble) classifications. This may be expected, as highly soluble drugs are more likely to show equivalence than BCS class 2 drugs. This is further supported by the U.S. FDA guidelines on waiver of *in vivo* BA and BE studies for immediate release solid oral dosage forms based on BCS class 1 and 3 system and also it is mentioned that the BCS-based biowaivers are not applicable for NTI drugs (and products designed to be absorbed in the oral cavity) (113, 114).

**Fig. 1:**
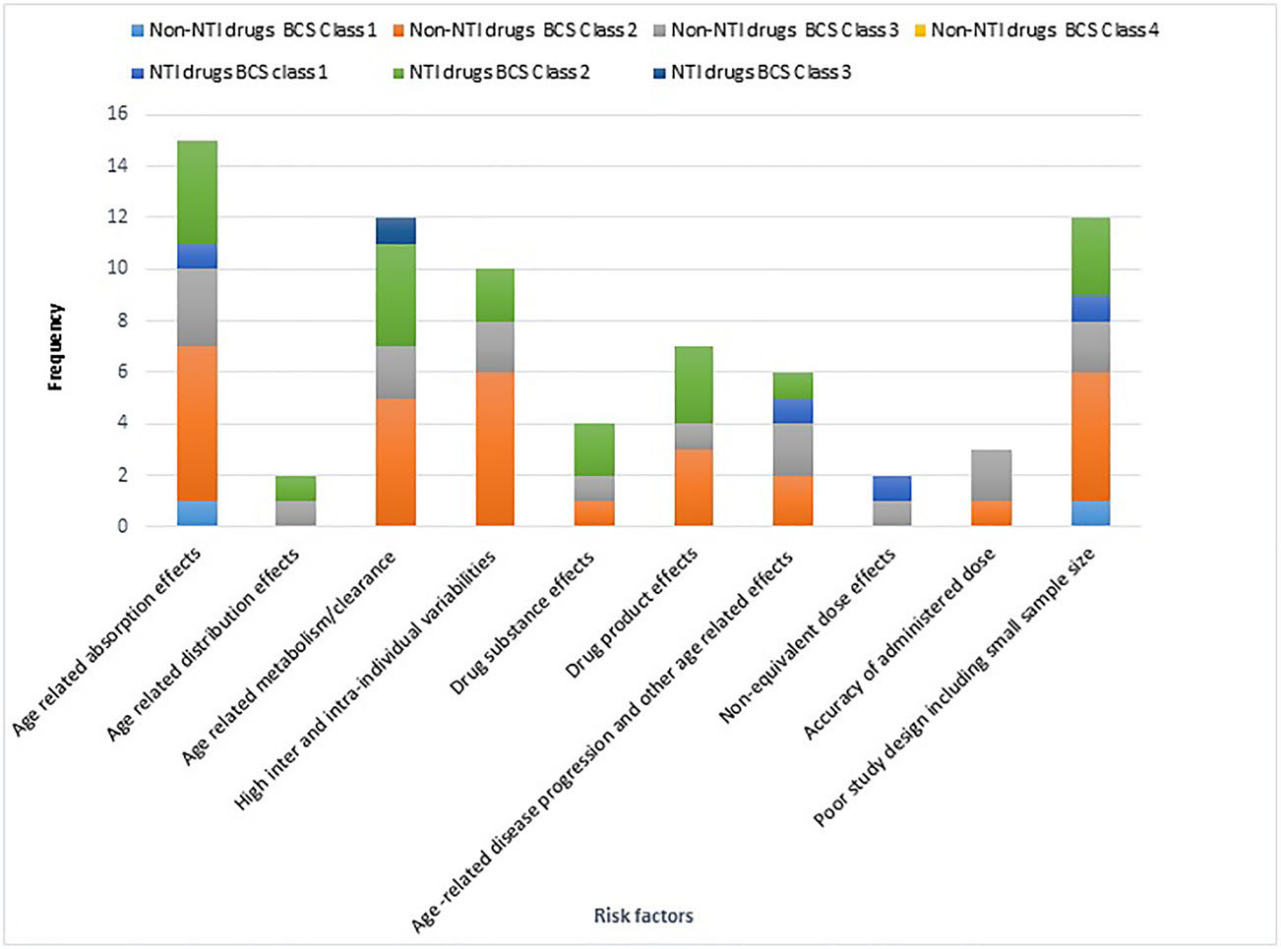
Bar chart showing the number of clinical trials involving non-NTI or NTI drugs under each risk factors analyzed

A comparison of the relative frequency risk factors for the studies is presented in Fig. 1. These are shown based on their pediatric BCS and non-NTI and NTI classification to identify where risks were most prevalent. Fig. 1 demonstrates that BCS class 2 drugs occupy the largest percentage of the DRBA cases where risk factors of age-related absorption effect, drug substance/drug product effect, and high inter- and intra-variabilities are identified.

## DISCUSSION

From Fig. 1, it is evident that age-related absorption risk factors for DRBA were mainly attributed to BCS class 2 non-NTI drugs (rifampicin, indinavir, efavirenz, pyrimethamine, lopinavir, ritonavir) and NTI drugs (e.g., carbamazepine, cyclosporin, phenytoin, and tacrolimus). The low solubility of BCS class 2 drugs at the site of absorption may lead to erratic absorption of NTI drugs. Moreover, only BCS class 2 (e.g., phenytoin) and BCS class 3 (e.g., lamivudine) drugs were identified as having DRBA risks due to age-related distribution effects. BCS class 2 drugs were observed at higher frequency for both non-NTI and NTI drugs for age-related metabolism risk factors. Interestingly, not a single case study or risk factor that related to excretion was found in the pediatric population.

Interestingly, drug substance-related risk factors were observed most often in BCS class 2 (non-NTI (*n* = 1); NTI (*n* = 2)) drugs. The differences in BE results due to drug products are seen at the highest frequency with both BCS class 2 categories for NTI drugs (*n* = 3 for BCS class 2). Moreover, both NTI and non-NTI BCS class 2 and 3 drugs showed DRBA due to disease progressions and other disease-related effects. Surprisingly, BCS class 1 NTI drugs (e.g., levothyroxine) despite having good solubility and permeability were also found to result in DRBA in a pediatric population although in this study, the claim of a difference in bioequivalence was based on the pharmacodynamic endpoint (thyroid-stimulating hormones) with smaller observed differences in the levothyroxine exposure. This is further supported by literature studies conducted for levothyroxine products in healthy or diseased adults (111, 115), and it is worth noting that the evidence of DRBA is not specific to the pediatric population. The poor stability of levothyroxine sodium tablets has been highlighted as they have been recalled many times since their 1955 introduction to the US market (116).

BCS class 2 (*n* = 6 for non-NTI and *n* = 2 for NTI drugs) and BCS class 3 (*n* = 2 for non-NTI) contributed notably for the observed inter- and intra-individual variations resulting in DRBA. The reason for inter- and intra-individual variations could be due to the physiological complexity of the GI tract and the physicochemical properties of the drug substance. The inter- and intra-individual variations in PK parameters are higher in pediatric population as compared to adults (92) due to the influence of growth, maturation, diurnal variations, pharmacogenetic reasons, the pre-systemic metabolism of the drug substance, etc. (48, 61, 117). The variations in PK parameters could be minimized by increasing the study power to > 80% and conducting a two-way, crossover single dose study (118). Our results indicated that low sample size and poor study design contributed as risk factors for DRBA results.

The collated risk factors resulting in DRBA will need to be further evaluated but could potentially serve as checkpoints during innovative pediatric formulation development, optimization of the prediction of a pediatric dose, and the extrapolation of BE results based on the clinical studies in adults. However, it should also be borne in mind that DRBA due to the aforementioned risk factors does not mean therapeutic inequivalence of pediatric medicines (119). If the final or to-be-marketed pediatric formulation is used in the pivotal pediatric clinical study for an innovator product, a BE/relative BA study for this product is not necessary. Any change to the formulation utilized in the pivotal study may trigger a clinical bridging study. It was not possible to generate sufficient data to stratify findings into subsets of the pediatric population (neonates, infants, children, and adolescents), yet this would be useful and additional data is needed to fully understand how risks of DRBA change with age within the pediatric population. Further work is warranted to also compare the magnitude of differences observed for the BE or relative BA data identified from the pediatric population with similar data from the adult population to fully evaluate the limitations of using adult data to predict the PK in pediatric populations.

## CONCLUSION

A database containing clinical studies on BE or relative BA in pediatrics has been developed and putative risk factors resulting in different relative BA are summarized. Only two publications were found that claimed to contain failed bioequivalence studies in a pediatric population. The vast majority of pediatric data comes from relative bioavailability studies of different formulations. Analysis of the developed database has highlighted that particular care is needed for BCS class 2 drugs when assessing BE in pediatrics. Additional work is warranted to use *in vitro* and *in silico* models for evaluating subtle changes in GI physiology that can affect the absorption of drugs in pediatric populations, particularly GI volume, motility, and transit times.

## Supplementary Information

ESM 1 (XLSX 314 kb)

ESM 2 (DOCX 135 kb)

## Declarations

### Disclaimer

The opinions expressed in this article are those of the authors and should not be interpreted as the position of the U.S. FDA and the cases being presented do not imply FDA’s endorsement of the products.
