# Supplementary material for: Development of a Pediatric Relative Bioavailability/Bioequivalence Database and Identification of Putative Risk Factors Associated With Evaluation of Pediatric Oral Products
Source: AAPS J. 2021 Apr 21;23(3):57. doi: 10.1208/s12248-021-00592-y (PMC8060189; doi:10.1208/s12248-021-00592-y)
Supplement: Supplementary file 2 — (DOCX 135 kb) [file 12248_2021_592_MOESM2_ESM.docx]

**Development of a pediatric relative bioavailability/bioequivalence database and identification of putative risk factors associated with evaluation of pediatric oral products**

Gopal Pawar^1*^, Fang Wu^2*^, Liang Zhao^2^, Lanyan Fang^2^, Gilbert J. Burckart^3^, Kairui Feng^2^, Youssef M Mousa^2^, Franci Naumann^1^, Hannah K. Batchelor^4*^

^1^School of Pharmacy, Institute of Clinical Sciences, University of Birmingham, Edgbaston, B15 2TT, UK

^2^Division of Quantitative Methods and Modelling, Office of Research and Standard, Office of Generic Drug Products, Center for Drug Evaluation and Research, United States Food and Drug Administration, Silver Spring, MD 20993.

^3^Office of Clinical Pharmacology, Office of Translational Science, Center for Drug Evaluation and Research, United States Food and Drug Administration, Silver Spring, MD 20993.

^4^Strathclyde Institute of Pharmacy and Biomedical Sciences, University of Strathclyde, 161 Cathedral Street, Glasgow G4 0RE

*Corresponding authors: Prof Hannah K Batchelor,

Professor in Pharmaceutics

Strathclyde Institute of Pharmacy and Biomedical Sciences, University of Strathclyde, 161 Cathedral Street, Glasgow G4 0RE

[Hannah.batchelor@strath.ac.uk](mailto:Hannah.batchelor@strath.ac.uk)

* [G.Pawar@bham.ac.uk](mailto:G.Pawar@bham.ac.uk)

* Fang.Wu@fda.hhs.gov

**Supplementary sheet 2**

**Table showing 79 clinical studies containing information on study details, potential risk factors resulting in bioinequivalence and referenced sentence from the particular study**.

Note that there are 84 rows with multiple color codings in the table as some of the studies have mentioned multiple putative risk factors.

Color coding indications- Blue- Absorption or Distribution or Metabolism/clearance related risk factors; Yellow- Drug substance and or drug product effects; Green (80% lighter)- Age- related disease progression and other disease related effects; Orange- Non-equivalent dose and accuracy of administered dose; Gray- Inter and intra individual variabilities; Green (40% lighter)- Poor study design including small sample size

|  | **Drug** | **Citation** | **Study details** | **Study Population** | **Test product** | **Reference product** | **Potential risk factors resulting in BIE** | **Referenced sentence from literature** |
| --- | --- | --- | --- | --- | --- | --- | --- | --- |
| 1 | Cyclosporin | Crocker 1998  (33) | Relative BA study (Switched from Sandimmune to Neoral) | Stable kidney transplants (n=22);11 >1 year post-transplant (average of 5.7 years); 9 were <1 year post-transplant | Microemulsion (Neoral) | Corn oil base (Sandimmune) | Absorption; Metabolism or clearance | In all children Neoral was rapidly absorbed and produced a high peak level of within 1 to 2 hours after dosing, compared with the levels achieved with Sandimmune  CyA, on entrance into the gastrointestinal tract and during its subsequent metabolism, gives rise to several metabolites, such a cyclosporin G, etc. Little is known about the changes in serum proﬁles of these metabolites. |
| 2 | Cyclosporine | Fu 1997  (46) | Relative BA study | Pediatric patients with lupus nephritis (n=10);9-14 years; Single oral- 5 mg/kg; Single dose; Fasted and fed | Cyclosporin A, Neoral, (Sandoz) | CsA capsules | Absorption | Results suggest that CsA Neoral has improved absorption and bioavailability. |
| 3 | Cyclosporine | Acott 2006  (32) | Relative BA study | Pediatric renal transplant patients (n=22-31) ;CYA group- 11.3 ± 1.2 years, MEC group - 8.6 ± 0.9 years | MEC (microemulsified galenic formulation of CsA). | CYA (oil-based predecessor) | Absorption  Metabolism | MEC is a microemulsion formulation of CsA that has been reported to have better absorption characteristics than CYA. MEC was reported to have a higher bioavailability than CYA in children, which is similar in magnitude to that reported for adults. The differences in required dosage administered likely reflects enhanced absorption of MEC compared to CsA because of excipient effects on drug transport or metabolism. |
| 4 | Cyclosporine | Krmar 1996  (93) | Relative BA study; non- randomized study ; stable transplant young patients study ; | Pediatric renal transplant patients (n=12);13.9 years (median age) | Cyclosporin A, Neoral, (Sandoz) capsules | solution | Absorption | Neoral exhibits a better and more predictable pattern of intestinal absorption |
| 5 | Phenytoin | Fukuyama 1982  (34) | Relative BA studies | Epileptic patients (n=24); 7-11 years | 24 were given Phenytoin Powder | 18 received Hydantol Tablet | Absorption;  Metabolism or clearance | Glazko et al demonstrated the fact that Sodium salt type is more easily absorbed from the intestinal tract than free acid type, and suggested that this is probably because sodium salt becomes even finer particles in the digestive tract. The absorption rate of PHT and its metabolic turnover varies by individual and age. |
| 6 | Cyclosporine | Dunn 1997  (96) | Relative BA study; open-label, multicenter, randomized crossover trial. | Pediatric maintenance liver transplant patients  n=31 patients  group 1, ages 1-5 years; group 2, ages 6-17 years | Test- new formulation, cyclosporine for microemulsion (CsA-ME) | Reference- original formulation (CsA) | Absorption | Previous studies have shown increased absorption and increased bioavailability of cyclosporine in pediatric liver transplant recipients treated with CsA-ME as compared with CsA. Therefore, the increased bioavailability of cyclosporine seems to be a direct consequence of the increased absorption of the drug.  Further, the consistency of the bioavailability of cyclosporine from CsA-ME as a function of age will help provide sustained immunosuppression even in very young liver transplant recipients. |
| 7 | Carbamazepine | Camfield 1989  (60) | Relative BA study | Epileptic children receiving chronic monotherapy CBZ (n =44 (23 boys, 21 girls); aged less than 6 years; Single dose; fasted | Tegretol Chew tabs | Tegretol | Absorption; metabolism or clearance | It is unclear whether such variations are related to fundamental differences in the way the two preparations are absorbed and/or metabolized by these individual patients. It is possible that patients on the same formulation of CBZ everyday have great variation of peak levels depending on diet or other factors. |
| 8 | Cyclosporine | Alvarez 2000  (104) | Relative BA study; Prospective multicenter randomized double-blind; Single dose pediatric study | Pediatric liver transplant recipients (n=32); 17 received Neoral and 15 received SIM. Neoral: 9 patients with mean age of 11.6±5.6 mo. SIM: 10 patients with a mean age of 12.5±6.3 months; 32 patients; 17 received Neoral and 15 received SIM. | Neoral | Sandimmune (SIM) | Absorption | In the very young children (younger than 2 years of age), Neoral resulted in significantly greater bioavailability and exposure than SIM which may be clinically important. Fasting resulted in greater exposure of Neoral over the 3 hr of the fasting study at day 23 as indicated by a higher Cmax and AUC0–3. This was not seen in the SIM group. Absorption of SIM was variable and was delayed in several patients (data not shown). Due to this variability it is possible that a higher AUC may have been observed if the PK study was of longer duration. Increased bioavailability with Neoral has been seen in other studies on paediatric patients including renal and liver transplant recipients |
| 9 | Cyclosporine | Portman 2000  (62) | Relative BA study; CsA GelCaps (Sandimmune) twice a day had been converted to CsA microemulsion (Neoral) on the same dose; single dose | Pediatric renal transplant recipients (n=14); 2.8–19.4 years | CsA microemulsion (Neoral) | CsA GelCaps (Sandimmune) | Absorption; metabolism or  clearance | The oral clearance (Cl/f) was significantly lower after the switch to Neoral, with reduced inter- and intraindividual variability. This allows for interesting extrapolations about the absorption of Neoral. The clearance of CsA is supposedly the same with Sandimmune and Neoral, as the pharmacologically active component CsA, which is detected in the blood by therapeutic drug monitoring, is identical for both drugs. For this reason, the variations in Cl/f after the conversion to Neoral are an expression of variations in absorption (f), as clearance (Cl) remains constant. Consequently, lower oral clearance with Neoral means higher absorption of Neoral, as previously reported. However, in addition, the lower inter- and intra-individual variability of Cl/f means lower variability in absorption. |
| 10 | Cyclosporine | Kelles 1999  (105) | Relative BA study | Stable renal allografts (n=25); mean age 14.1 years | Neoral | Sandiimmune (SIM) | Absorption | Our results show that NEO exhibits a better pattern of oral absorption and improved bioavailability than SIM |
| 11 | Phenytoin | Hodges 1986  (61) | Relative BA study | Epileptic children (n=30;18 boys, 12 girls) mean 9.6 years | 50mg capsules from Parke-Davis (PD) and 50mg tablets from Boots (B) and from Evans € | NA | Absorption  Metabolism | Phenytoin demonstrates dose-related saturation, which generally is considered to occur at a serum concentration of 10 mg/l, i.e. the elimination half-life increases as liver enzyme metabolic pathways are saturated. The effect is an extended duration of phenytoin in the body and consequently accumulation to a higher serum level on chronic administration. Further, as protein-binding sites are fully used, small dose changes can  significantly affect the level of ‘free’ (and therefore clinically relevant) phenytoin available |
| 12 | Cyclosporin | van Mourik 1999  (85) | Relative BA study | Stable Pediatric Liver Transplant Recipients (n=8); 1.2-12 years | Neoral oral solution | Sandimmune oral solution | Absorption | Neoral is absorbed better than Sandimmune. In conclusion, Neoral is more rapidly and consistently absorbed than Sandimmune in children after liver transplantation. |
| 13 | Cyclosporine | Melter 1997  (38) | Relative BA study (Switch from Sandimmune to Neoral) | Pediatric long-term liver transplant recipients; 38 children (19 girls, 19 boys); 2-12.3 years | Microemulsion (Neoral) | Corn oil base (Sandimmune) | Absorption | In conclusion, the main difference between Neoral (NEO) and Sandimmune (SIM) is their absorption. In NEO, it is greater, as reflected in a shorter Tmax  higher Cmax and higher AUC; there is also a strong relationship between AUC and corresponding single-point blood concentrations. These guarantee a better predictability of systemic drug exposure especially from C2h, and permit less drug monitoring and fewer dose adjustments. It has been suggested that NEO converts all so-called poor absorbers of SIM to good absorber status. |
| 14 | Emtricitabine | Wang 2004  (44) | Relative BA study; Phase I, open-label, nonrandomized, dose-escalation, multicenter study | HIV-infected children; Cohort 2 (n=2); Cohort 3 (n=8); Cohort 4 (n=8); Cohort 5 (n=7); < 18 years | Oral solution Emtricitabine | Capsule Emtricitabine | Absorption | It is likely that the gastrointestinal transit time of the solution formulation is shorter than that of the capsule formulation, thereby reducing the mucosal contact time for Emtricitabine to be absorbed. |
| 15 | Lamivudine | Kasirye 2012  (8) | Relative BA study; Two-period, crossover, open-label study | HIV infected children (n=19) (10 < 12 kg); fasted | Scored tablets of combivir (coformulated zidovudine+ lamivudine ) and abacavir | 10 ml (100mg)- Zidovudine Oral solution, 5 ml (100 mg) abacavir oral solution, 5 ml (100mg) lamivudine oral solution | Absorption | This finding suggests that the formulation–related effect observed in children but not in adults may be in some manner related to the age–dependent differences in absolute bioavailability observed for these two drugs. Other possible hypotheses for formulation–dependent differences in relative bioavailability between children and adults are (i) differential effects of increased gastrointestinal transit time or stomach pH in children as compared to adults and (ii) the solution may lead to very high local concentrations of active components in children resulting in saturation of gastrointestinal membrane transporters. |
| 16 | Indinavir | Mueller 1998  (41) | Relative BA study; Phase I/II study; progressive HIV disease; n=54 | HIV disease (n=54); Median 9.7 (3.1 to 18.9) years | Capsule formulation (sulfate salt dry-filled capsules) | Free-base suspension (jet milled suspension) | Absorption | Comparative analysis of the PK obtained after the same dose of the liquid formulation and the capsule formulation revealed not only a marked interpatient variability but also a substantially lower absorption of the indinavir free based suspension |
| 17 | Hydrocortisone | Merke 2001  (39) | Relative BA study | Classic form of 21 hydroxylase; n=19 (13 boys, 6 girls); 9-12 years | Liquid hydrocortisone formulation (Cortef: 10 mg/ml) | Hydrocortisone tablets (dose was reduced to ~ 10%) | Absorption | Differences in bioavailability for  Cortef suspension and hydrocortisone tablets; hydrocortisone without ester form have higher absorption rate than the tablet form |
| 18 | Efavirenz | ter Heine 2008  (43) | Relative BA study | HIV-1 infected children (n =33; females =16); median age of 33 children- 6.5 years (0.9-19 years) | Efavirenz Liquid formulation (30 mg/ml) | Capsules/tablets (200, 250, 300, 350, 400 & 600 mg) | Absorption  Metabolism or clearance | Physiological difference b/w healthy adults and HIV-infected children that could influence drug absorption.  Cl/F is the major determinant of decreased efavirenz exposure in children. |
| 19 | Cyclosporine | Medeiros 1998  (37) | Relative BA study; Randomized, double blind, cross-over design | 10 children with end-stage renal disease (ESRD); < 18 years | Neoral | Sandimmun | Absorption | Oral CsA used to be available only as an oily oral solution until recently (Sandimmun, Sandoz; SAN). CsA intestinal absorption is erratic with the formulation since it depends on bile secretion. |
| 20 | Cyclosporine | Kabasakul 1997  (36) | Relative BA study | 9 renal transplant recipients (range 4.8–10.9 years) | Neoral | Sandimmun | Absorption; metabolism | Increased frequency of postoperative ileus, short gut length, higher gut motility and faster metabolic rate may also directly influence CYA absorption and bioavailability. |
| 21 | Cyclosporine | Bokenkamp 1995  (45) | Relative BA study | 10 renal transplant recipients (median age 14.9 years) | Neoral | Sandimmun | Absorption | Absorption studies showed Sandimmun malabsorption in six of the ten children. |
| 22 | Lopinavir and Ritonavir | Best 2011  (57) | Relative BA study; Randomized, open-label, cross-over study | HIV-infected children (n=12); 10–16 years, Fasted | Crushed tablets | Whole 200/50 mg lopinavir/ritonavir tablets. | Absorption;  Metabolism or clearance; Volume of distribution | After the intake of the Kaletra tablets, lopinavir (LPV) and ritonavir (RTV) plasma concentrations rose faster with crushed tablets immediately after administration, possibly because of easier absorption due to larger surface area of drug particles, but fell off as time progressed leading to a lower total exposure in crushed compared with whole tablets. As a result, the maximum plasma concentrations of both LPV and RTV were lower with crushed tablets.  The potential changes in the CYP450 based metabolism in the gut and intracellular drug transport may be responsible for the observed changes in AUC for lopinavir and ritonavir. |
| 23 | Lamivudine/Nevirapine | Chokephaibulkit 2011  (63) | Relative BA study; Phase I/II two-arm randomized open-label multiple dose PK cross-over study | HIV-1 infected children (n= 42 (18 males); ≥5 months to <13 years of age | FDC tablets | Oral solution | Metabolism or  Clearance | Genetic polymorphisms in drug metabolizing enzymes and transporters have been shown to contribute towards antiretroviral inter-individual drug variability. Nevirapine (NVP) is extensively metabolized via CYP enzymes, in particular the isoenzymes CYP2B6 and CYP3A. In 126 children from PACTG 366 and 377 cohorts, the CYP2B6 516G>T single nucleotide polymorphism was associated with slower NVP oral clearance and an improved immunological response. In the current study, the frequency of the T/T alleles was 10%, which is consistent with other reports in the Thai population [35], and was associated with significantly higher NVP exposure; however, there was no evidence of increased toxicities among children with this genotype  This could be explained by the faster NVP clearance in younger children  May be faster clearance in children; NVP exposure increased with age |
| 24 | Rifampicin | McIlleron 2016  (77) | Relative BA study | South African tuberculosis children (n=146); 0.2–10.2 years | A granulate preparation of RMP for suspension (92 received Eremfat) | 54 received R-Cin formulations | Absorption | We therefore hypothesise that the differences observed in bioavailability are due to the mixture of polymorphic forms of RMP in R-Cin that was not favorable for absorption. The water solubility of RMP is reported to vary eight-fold depending on the crystalline state of the material, altered particle size affects solubility and altered solubility is likely to affect bioavailability. However, the relationship between solid-state RMP, dissolution and bioavailability characteristics is poorly understood. |
| 25 | Stavudine Lamivudine Nevirapine | Vanprapar 2010  (67) | Relative BA study; Phase I/II, 2 stage, 2 arm, randomized, open-label | HIV-1 infected Thai children (n=34); ≥6 months to <13 years of age | Chewable tablets | Oral solutions | Metabolism or clearance | NVP clearance to be more rapid in younger children |
| 26 | Tacrolimus | Reding 2002  (81) | Relative BA study | Liver transplant (n=15); 0.4–10.6 years; two daily dose | Oral suspension of tacrolimus (OST) | Tacrolimus capsules (TC) | Absorption | The lower BA observed for the  oral suspension does not appear to be secondary to an increased hepatic first-pass effect; consequently,  an incomplete solubility or absorption of the OST could be hypothesized, which would  explain the delay of Tmax and lower AUC observed |
| 27 | Nevirapine | Ellis 2007  (65) | Relative BA study | African HIV-infected children (n=56); aged 8 months to 18 years; Once and twice daily | Triomune tablets (stavudine+lamivudine+nevirapine). | Either whole (200 mg) or divided into quarter (50 mg Nevirapine), half (100 mg ) or three quarter (150 mg) (Accg to surface area WHO guidelines) | Metabolism or clearance | Nevirapine is metabolized more rapidly in younger children; Our results suggested that nevirapine concentrations are reduced in stunted children, which have higher surface for their weight. |
| 28 | Levothyroxine | Carswell 2013  (26) | BE study; 16-week, open-label, randomized, controlled, crossover trial | Children with severe congenital hypothyroidism (n=31); 3-18 years | AB-rated generic formulation | L-T4 (Synthroid) | Absorption | Because infants feed frequently, it is often not possible to administer l-T4 in a fasting state, thereby magnifying potential differences in dissolution properties between l-T4 formulations |
| 29 | Phenytoin | Matsukura 1984  (40) | Relative BA study; cross-over study | Pediatric patients and healthy adults (n=93 children); n= 5 adults (25-32 years) | Aleviatin powder | Hydantol tablet | Absorption; Volume of distribution, protein binding | Other factors such as apparent volume of distribution, protein binding and bioavailability should be considered for pediatric PK profile |
| 30 | Sulfadoxine (SDX)-Pyrimethamine (PYR) | Salman 2011  (42) | Relative BA study | Malaria (n=70); 2 to 13 months | SDX/PYR  (Fansidar); All drugs were crushed and mixed with either water or breast milk | Double dose | Absorption; Metabolism or clearance | There was a 32% reduction in the relative BA of SDX when the dose was doubled, possibly due to saturation of absorption. A limited maturation of elimination processes is likely to play a role in the longer *t*1/2 and higher AUC observed for both drugs. Indeed, we found evidence of a slower maturation of these processes for SDX than PYR. |
| 31 | Tacrolimus | Jacobo-Cabral 2014  (35) | BE study | Renal transplant recipients; Prograf group (n=29); Limustin (n=9); Prograf group- 12-16 years; Limustin-14.5-17 years; Oral twice a day; Fasted | Limustin | Prograf | Absorption | The reduced AUC and Cmax values with Limustin in children are likely the outcome of a low amount of drug dissolved in the GI lumen limiting the extent of absorption. |
| 32 | Tacrolimus | \| Vondrak 2018  (64) \| \| --- \| | Relative BA study; Phase 2, parallel-group, multicenter, open-label, 4-week study | De novo pediatric patients undergoing primary kidney, liver, or heart transplantation; 15 patients received PR tacrolimus (kidney, n = 10; liver, n = 3; heart, n = 2), and 18 received the IR formulation (kidney, n = 10; liver, n = 5; heart, n = 3); Single or twice | Prolonged-release Tacrolimus | Immediate release Tacrolimus | Metabolism or clearance | Tacrolimus clearance is known to be higher in younger children. |
| 33 | Carbamazepine | Eeg-Olofsson 1990  (66) | Relative BA study; Open, controlled, within-patient study, Fasted | Epileptic children (n=25); 4-13 years | Tegretol SR | Tegretol CBZ | Metabolism or clearance | The difference between children and adults is most probably explained by the more rapid rate of metabolism in children compared with adults. |
| 34 | Cyclosporine | Acott 2006  (32) | Relative BA study | Pediatric renal transplant patients (n=22-31); CYA group- 11.3 ± 1.2 years, MEC group - 8.6 ± 0.9 years | 1.7 mg/kg/dose for MEC (microemulsified galenic formulation of CsA). | CYA (oil-based predecessor) | Drug product effects | The predominant difference in formulation is at the level of absorption; the carrier base is the likely cause of differences in CsA bioavailability |
| 35 | Cyclosporine | Medeiros 1998  (37) | Relative BA study; randomized, double blind, cross-over design | 10 children with end-stage renal disease (ESRD);< 18 years | Neoral | Sandimmune | Drug product effects | Our results indicate that, in children with ESRD, NEO yielded CsA AUC and Cmax values that were 90% and 130% higher, respectively than those observed with SAN. |
| 36 | Cyclosporine | Kabasakul 1997  (36) | Relative BA study | 9 renal transplant recipients (range 4.8–10.9 years) | Neoral | Sandimmune | Drug product effects | Variable concentrations of CYA may contribute to the higher allograft loss from rejection observed in young children with renal transplants. |
| 37 | Cyclosporine | Bokenkamp 1995  (45) | Relative BA study | 10 renal transplant recipients (median age 14.9 years) | Neoral | Sandimmun | Drug product effects | Large increase in both  Cmax and AUC after switching to Neoral |
| 38 | Efavirenz | ter Heine 2008  (43) | Relative BA study; Open label study | HIV-1 infected children (n =33; females =16); median age of 33 children- 6.5 years (0.9-19 years) | Efavirenz Liquid formulation (30 mg/ml) | Capsules/tablets (200, 250, 300, 350, 400 & 600 mg) | Drug product effects | Drug formulation had a large effect on the BA of efavirenz. Relative BA of the oral liquid compared with tablets or capsules was only 46.6% |
| 39 | Hydrocortisone | Merke 2001  (39) | Relative BA study | Classic form of 21 hydroxylase; n=19 (13 boys, 6 girls); 9-12 years | Liquid hydrocortisone formulation (Cortef: 10 mg/ml) | Hydrocortisone tablets (dose was reduced to ~ 10%) | Drug product effects  Drug Substance effect | Tablet is hydrocortisone and suspension is hydrocortisone cypionate; both are not chemically equivalent; reformulation of the suspending agent might have effected drug concentrations or the extent of compliance with shaking instructions. |
| 40 | Rifampicin | McIlleron 2016  (77) | Relative BA study | South African tuberculosis children (n=146); 0.2–10.2 years | A granulate preparation of Rifampicin (RMP) for suspension (92 received Eremfat) | 54 received R-Cin formulation | Drug substance effects | The authors hypothesize that the differences observed in BA are due to the mixture of polymorphic forms of RMP in R-Cin that was not favourable for absorption. Low RMP concentrations attributed to formulation effects. |
| 41 | Cyclosporine | Melter 1997  (38) | Relative BA study (Switch from Sandimmune to Neoral) | Pediatric long-term liver transplant recipients; 38 children (19 girls, 19 boys); 2-12.3 years | Microemulsion (Neoral) | Corn oil base (Sandimmune) | Drug product effects | This study revealed an increased and more consistent absorption of cyclosporin from the NEO formulation |
| 42 | Indinavir | Mueller 1998  (41) | Relative BA study; phase I/II study; progressive HIV disease; median 9.7 (3.1 to 18.9) years; n=54 | HIV disease (n=54); Median 9.7 (3.1 to 18.9) years | Capsule formulation (sulfate salt dry-filled capsules) | Free-base suspension (jet milled suspension) | Drug product effects | Capsule formulation revealed not only a marked interpatient variability but also a substantially lower absorption of the indinavir free based suspension |
| 43 | Phenytoin | Fukuyama 1982  (34) | Relative BA studies | Epileptic patients (n=24); 7-11 years | 24 were given Phenytoin Powder | 18 received Hydantol Tablet | Drug substance effects; drug product effects | Glazko et al demonstrated the fact that Sodium salt type is more easily absorbed from the intestinal tract than free acid type, and suggested that this is probably because sodium salt becomes even finer particles in the digestive tract. |
| 44 | Phenytoin | Matsukura 1984  (40) | Relative BA study; cross-over study | Children patients and healthy adults; 93 children patients; 5 healthy adults (25-32 years); Single and multiple dose; dose- 7 mg/kg | Aleviatin powder | Hydantol tablet | Drug substance effects; Drug product effects | BA was greater in the preparation with the smaller particle size and more rapid in *invitro* dissolution; Rel BA increased with increasing age (age dependent effect on drug absorption) |
| 45 | 6-Mercaptopurine | Tolbert 2017  (82) | Relative BA study; Randomized two-way, crossover study of two cohorts; prospective, single-site pediatric pharmacokinetic and BA study comparing two oral liquid 6-MP formulations (5 or 50 mg/ml) | Acute lymphoblastic leukemia (ALL) children (n=22); 6–17 years; Fasted | 2 extemporaneously prepared liquid formulations, 6-mercaptopurine (6-MP) liquid | Marketed tablet Purixan R | Drug product effects | Differences in formulation constituents, viscosity, and potentially 6-MP concentration likely contribute to the variability observed in systemic drug availability from liquid formulations. |
| 46 | Tacrolimus | Jacobo-Cabral 2014  (35) | BE study | Renal transplant recipients; Prograf group (n=29); Limustin (n=9); Prograf group- 12-16 years; Limustin-14.5-17 years; Oral twice a day; Fasted | Limustin | Prograf | Drug substance effects and drug product effects | Tacrolimus is a class II drug in the biopharmaceutical classification system. That is, it exhibits low solubility and high permeability. Limustin® exhibits pharmaceutical characteristics dissimilar to the innovator that likely explain the reduced tacrolimus exposure. |
| 47 | Levothyroxine | Carswell 2013  (26) | BE study; 16-week, open-label, randomized, controlled, crossover trial | Children with severe congenital hypothyroidism (CH)(n=31); 3-18 years | AB-rated generic formulation | l-T4 (Synthroid) | Age- related disease progression and other disease related effects | Synthroid and an AB-rated generic L-T4 are not bioequivalent for patients with severe hypothyroidism due to CH, probably because of diminished thyroid reserve.  A third and more likely reason for our results was therefore diminished thyroid hormone reserve in the patients with CH (36). In support of this possibility, most of the patients with CH had a thyrosis or severe thyroid dysgenesis on imaging, and as noted above, patients with CH required a higher l-T4 dose for control of their hypothyroidism, consistent with findings in adults with severe hypothyroidism |
| 48 | Stavudine Lamivudine Nevirapine | Mukherjee 2014  (86) | Relative BA study | HIV-infected children; N=79 | FDCs generic drugs-Stavudine, lamivudine, nevirapine in the ratio of 6:30:50mg | NA | Age- related disease progression and other disease related effects | Doses of stavudine and lamivudine appeared to be lower in children older than three years, and if they were stunted. Subtherapeutic nevirapine levels were observed in 35% of children, especially the younger ones. Also malnourished state like stunting and the CYP2B6 GG or GT genotype were found to be predictors for low nevirapine concentration |
| 49 | Cyclosporin | van Mourik 1999  (85) | Relative BA study | Stable Pediatric Liver Transplant Recipients (n=8); 1.2-12 years | Neoral oral solution | Sandimmune oral solution | Age- related disease progression and other disease related effects | The absorption of Sandimmune is affected by the type of biliary anastomosis and underlying disease. In this study, the 6 patients with Rouxen-Y loop biliary anastomosis showed greater improvement in Neoral absorption than the 2 patients with duct-to-duct biliary anastomosis, suggesting that Neoral improves bioavailability in this particular group. Impaired gastrointestinal function, e.g., in cystic fibrosis where intestinal absorption is dependent on pancreatic enzyme supplementation, may also affect Sandimmune bioavailability |
| 50 | 6-Mercaptopurine | Tolbert 2017  (82) | Relative BA study; Randomized two-way, crossover study of two cohorts; prospective, single-site pediatric PK and BA study comparing two oral liquid 6-MP formulations (5 or 50 mg/ml) | Acute lymphoblastic leukemia (ALL) children (n=22); 6–17 years; Fasted | 2 extemporaneously prepared liquid formulations, 6-mercaptopurine liquid | Marketed tablet Purixan R | Age- related disease progression and other disease related effects | FDA regulation recommends that the efficacy data from adult population only be extrapolated to pediatric populations when the course of the disease and the effects of the drug are sufficiently similar in the two populations. In this case, the course of the disease in children differs from adults and the pharmacokinetic results would suggest that the populations differ with regard to drug disposition |
| 51 | Hydrocortisone | Merke 2001  (39) | Relative BA study | Classic form of 21 hydroxylase (n=19,13 boys, 6 girls) | Liquid hydrocortisone formulation (Cortef: 10 mg/ml) | Hydrocortisone tablets (dose was reduced to ~ 10%) | Non-equivalent dose | Smallest 5 mg tablet dose is difficult to achieve; reformulation of the suspending agent might have effected drug concentrations or the extent of compliance with shaking instructions. |
| 52 | L-Thyroxine | Cassio 2013  (98) | Relative BA study | Congenital hypothyroidism infants (n=42 infants (25 girls, 17 boys); < 37 weeks; Single dose; Fasted | Oral drops | Tablets | Non-equivalent dose | 1) Mistakes in administration (e.g. no of drops administered, volume of the mixed liquid), or the liquid form is more efficacious than the tablet, which has to be crushed, undergoing a process that might cause some loss of the drug. |
| 53 | Stavudine Lamivudine Nevirapine | Pollock 2009  (99) | Relative BA study | HIV-1-infected malnourished (12 (32%)) and normal children (25 (68%); 0–16 years | Triomune 30 (30 mg of stavudine, 150 mg of lamivudine and 200 mg of nevirapine) | All (n=37)-Nevirapine- 200 mg; ; Normal (n=25)-Nevirapine-250 mg; Stavudine-; malnourished- 125 mg; Lamivudine-11 mg/kg; Stavudine-2.2 mg/kg | Accuracy of administered dose | Nevirapine exposure was strongly related to dose administered and to age; Use of divided adult Triomunew30 tablets in treating young children results in signiﬁcant underdosing. |
| 54 | Nevirapine | Ellis 2007  (65) | Relative BA study | African HIV-infected children (n=56); aged 8 months to 18 years; Once and twice daily | Triomune tablets (stavudine+lamivudine+nevirapine). | Either whole (200 mg) or divided into quarter (50 mg Nevirapine), half (100 mg N) or three quarter (150 mg) (Accg to surface area WHO guidelines) | Accuracy of administered dose | Younger, smaller children who are more likely to receive quarter tablets and have higher surface area of their weight are at particular risk, with nearly half of those receiving quarter tablets either once or twice daily having subtherapeutic concentrations. |
| 55 | Cyclosporine | Krmar 1996  (93) | Relative BA study; Non- randomized study ; stable transplant young patients study | Pediatric renal transplant patients (n=12);13.9 years (median age) | Cyclosporin A, Neoral, (Sandoz) capsules | solution | Inter-individual variabilities | Large interindividual variability of CyA PK in this age group needs close monitoring |
| 56 | Hydrocortisone | Merke 2001  (39) | Relative BA study | Classic form of 21 hydroxylase (n=19, 13 boys, 6 girls); 9-12 years | Liquid hydrocortisone formulation (Cortef: 10 mg/ml) | hydrocortisone tablets (dose was reduced to ~ 10%) | Inter-individual variabilities | Interindividual variations in cortisol metabolism and ability to suppress androgens should be considered |
| 57 | Indinavir | Mueller 1998  (41) | Relative BA study; phase I/II study; progressive HIV disease; median 9.7 (3.1 to 18.9) years; n=54 | HIV disease (n=54); Median 9.7 (3.1 to 18.9) years | Capsule formulation (sulfate salt dry-filled capsules) | Free-base suspension (jet milled suspension) | Inter-individual variabilities | Marked interpatient variability |
| 58 | Cyclosporine | Fu 1997  (46) | Relative BA study | Pediatric patients with lupus nephritis (n=10);9-14 years; Single oral- 5 mg/kg; ; Single dose; Fasted and fed | Cyclosporin A, Neoral, (Sandoz) | CsA capsules | Inter-individual variabilities | CsA Neoral had improved absorption and bioavailability bud did not significantly reduce interindividual variability still exists |
| 59 | Lopinavir (LPV) Ritonavir .(r) | Best 2011  (57) | Relative BA study;  Randomized, open-label, cross-over study | HIV-infected children (n=12); 10–16 years; Fasted | Crushed tablets | Whole 200/50 mg lopinavir/ritonavir tablets. | Inter-individual variabilities | Higher variability of lopinavir/ritonavir in children and is possibly due to age, physical and sexual maturation and weight differences of the subjects |
| 60 | Cyclosporine | Melter 1997  (38) | Relative BA study (Switch from Sandimmune to Neoral) | Pediatric long-term liver transplant recipients; 38 children (19 girls, 19 boys); 2-12.3 years | Microemulsion (Neoral) | Corn oil base (Sandimmune) | Inter-individual variabilities | The clinical use of SIM is associated with highly variable PK parameters, especially following pediatric OLT  Several studies have shown an improved absorption and a more consistent bioavailability of cyclosporin from NEO than from SIM in adults and in pediatric kidney recipients |
| 61 | Efavirenz | Salem 2014  (94) | Relative BA study; Open-label phase I/II study | HIV-1-infected (n=96); cohort I 3 to 16 years old cohort II 2 months and 8 years old | Oral liquid formulations: suspension and solution (test) | Capsule formulation | Inter-individual variabilities | The liquid formulation BA relative to the capsule was found to increase with age to reach 90% of its mature value by the age of 8 years. The CYP2B6-G516T polymorphism decreased oral clearance. Similarly high variability has been estimated in other studies, as well and could be attributed to polymorphisms in metabolizing enzymes |
| 62 | 6-Mercaptopurine | Tolbert 2017  (82) | Relative BA study ; Randomized two-way, crossover study of two cohorts; prospective, single-site pediatric pharmacokinetic and BA study comparing two oral liquid 6-Mercaptopurine (6-MP) formulations (5 or 50 mg/ml) | Acute lymphoblastic leukemia (ALL) children (n=22); 6–17 years; Fasted | 2 extemporaneously prepared liquid formulations, 6-mercaptopurine liquid | Marketed tablet Purixan R | Inter- and intra-individual variabilities | High inter- and intraindividual variability of 6-MP pharmacokinetics (PK). |
| 63 | Lamivudine/  Nevirapine | Chokephaibulkit 2011  (63) | Relative BA study; Phase I/II two-arm randomized open-label multiple dose PK cross-over study | HIV-1 infected children (n= 42 (18 males); ≥5 months to <13 years of age | FDC tablets | Oral solution | Inter-individual variabilities | Genetic polymorphisms in drug metabolizing enzymes and transporters have been shown to contribute towards antiretroviral inter-individual drug variability. Nevirapine (NVP) is extensively metabolized via CYP enzymes, in particular the isoenzymes CYP2B6 and CYP3A. In 126 children from PACTG 366 and 377 cohorts, the CYP2B6 516G>T single nucleotide polymorphism was associated with slower NVP oral clearance and an improved immunological response. In the current study, the frequency of the T/T alleles was 10%, which is consistent with other reports in the Thai population, and was associated with significantly higher NVP exposure; however, there was no evidence of increased toxicities among children with this genotype  This could be explained by the faster NVP clearance in younger children |
| 64 | Carbamazepine | Camfield 1989  (60) | Relative BA study | Epileptic children receiving chronic monotherapy CBZ (n =44 (23 boys, 21 girls); aged less than 6 years; Single dose; fasted | Tegretol Chew tabs | Tegretol | Inter-individual variabilities | In five patients chewable carbamazepine (CBZ) produced higher peak CBZ levels while five had higher peaks with regular CBZ |
| 65 | Carbamazepine | Eeg-Olofsson 1990  (66) | Relative BA study; Open, controlled, within-patient study, Fasted | Epileptic children (n=25); 4-13 years | Tegretol SR | Tegretol CBZ | Interindividual  variations | Plasma concentration curves during treatment with the slow-release formulation showed significantly less variation over 24 hours than during treatment with the ordinary preparation, as measured by the fluctuation index |
| 66 | Cyclosporine | Portman 2000  (62) | Relative BA study; CsA GelCaps (Sandimmune) twice a day had been converted to CsA microemulsion (Neoral) on the same dose; single dose | Pediatric renal transplant recipients (n=14); 2.8–19.4 years | CsA microemulsion (Neoral) | CsA GelCaps (Sandimmune) | Intraindividual variabilities | After the switch to Neoral, study observed less inter- and intra-individual variability of AUC, the 2-h concentration, and the oral clearance |
| 67 | Cyclosporin | Crocker 1998  (33) | Relative BA study (Switch from Sandimmune to Neoral) | Stable kidney transplants (n=22);11 >1 year post transplant (average of 5.7 years); 9 were <1 year post transplant | Microemulsion (Neoral) | Corn oil base (Sandimmune) | Interindividual variabilities | Less interpatient absorption variability with the microemulsion formulation compared with the original corn oil preparation |
| 68 | Lopinavir/Ritonavir | Musiime 2014  (95) | Relative BA study; Open, randomized, phase I, 2-period crossover comparative bioavailability trial | HIV infected infants and Ugandan children;3 to < 12 months (cohort A); children aged 1–4 years (cohort B); children aged 4 to ,13 years (cohort C);infants/children were included in cohort A (n = 19)/B (n = 26)/C (n = 32) | Minitab Sprinkles | Syrup; tablets | Inter-individual variabilities | Variability in Lopinavir LPV/r  PK parameters was moderate to high with all formulations |
| 69 | Cyclosporine | Medeiros 1998  (37) | Relative BA study ; randomized, double blind, cross-over design | 10 children with end-stage renal disease (ESRD);< 18 years | Neoral | Sandimmune | Interindividual variability | We did not observe a reduction in interindividual variability, as suggested by Cooney et al., however our study was not carried out in transplant patients as theirs was, and the uremic milieu may be responsible for our inability to show reduction in interindividual variability. |
| 70 | Cyclosporine | Kabasakul 1997  (36) | Relative BA study | 9 renal transplant recipients (range 4.8–10.9 years) | Neoral | Sandimmune | Inter individual variabilities | Variable concentrations of CYA may contribute to the higher allograft loss from rejection observed in young children with renal transplants. |
| 71 | Cyclosporine | Dunn 1997  (96) | Relative BA study; open-label, multicenter, randomized crossover trial. | pediatric maintenance liver transplant patients  n=31 patients  group 1, ages 1-5 years; group 2, ages 6-17 years | Test- new formulation, cyclosporine for microemulsion (CsA-ME) | Reference- original formulation (CsA) | Inter and intra individual variabilities | The large inter- and intra-individual variability in cyclosporine absorption and bioavailability from the original formulation of cyclosporine (CsA*) has limited the ability to attain consistently optimum individual immunosuppression and has necessitated frequent blood monitoring to ensure that the CsA dosage is sufficient to maintain adequate trough cyclosporine levels. |
| 72 | Cyclosporine | Bokenkamp 1995  (45) | Relative BA study | 10 renal transplant recipients (median age 14.9 years) | Neoral | Sandimmun | Inter and intra individual variabilities | With Sandimmun, the large intraindividual and interindividual variability of CsA pharmacokinetic  parameters in kidney transplant recipients is well known |
| 73 | L-Thyroxine | Cassio 2013  (98) | Relative BA study | CH infants (n=42 infants (25 girls, 17 boys); < 37 weeks; Single dose; Fasted | Oral drops | Tablets | Poor study design including small sample size | Small sample of patients enrolled in a single center |
| 74 | Emtricitabine | Wang 2004  (44) | Relative BA study; Phase I, open-label, nonrandomized, dose-escalation, multicenter study | HIV-infected children; Cohort 2 (n=2); Cohort 3 (n=8); Cohort 4 (n=8); Cohort 5 (n=7); < 18 years old | Oral solution Emtricitabine (10 mg/ml) for the first two doses | Capsule Emtricitabine | Poor study design including small sample size | The small sample size and the nonrandomized, pilot study nature |
| 75 | Cyclosporin | van Mourik 1999  (85) | Relative BA profile study | Stable Pediatric Liver Transplant Recipients (n=8); 1.2-12 years | Neoral oral solution | Sandimmune oral solution | Poor study design including small sample size | Although the AUC was 25% higher with Neoral than with Sandimmune, this was not statistically significant for this small group of patients |
| 76 | Phenytoin | Hodges 1986  (61) | Relative BA study | Epileptic children (n=30;18 boys, 12 girls) mean 9.6 years | 50mg capsules from Parke-Davis (PD) and 50mg tablets from Boots (B) and from Evans € | NA | Poor study design | A longer study with more patients might reveal significant differences. |
| 77 | Rifampicin | McIlleron 2016  (77) | Relative BA study | South African tuberculosis children (n=146); 0.2–10.2 years | A granulate preparation of Rifampicin (RMP) for suspension (92 received Eremfat) | 54 received R-Cin formulations | Poor study design and small sample size | Our study was not designed to compare the bioavailability of the formulations used, and the estimates of the effect of formulation type on the bioavailability of RMP were limited by a relatively small sample size for accurate adjustment for potentially confounding factors such as age, weight and nasogastric tube use |
| 78 | Tacrolimus | Vondrak 2018  (64) | Relative BA study; Phase 2, parallel-group, multicenter, open-label, 4-week study | De novo pediatric patients undergoing primary kidney, liver, or heart transplantation; 15 patients received prolonged release tacrolimus (kidney, n=10; liver, n=3; heart, n=2), and 18 received the immediate-release formulation (kidney, n=10; liver, n=5; heart, n= 3); Single or twice | Prolonged-release Tacrolimus | Immediate release Tacrolimus | Poor study design and small sample size | Limitations- Patient numbers were small and, therefore, it was not possible to stratify the analyses by organ type. Study did not include any children below 4 years of age |
| 79 | Tacrolimus | Reding 2002  (81) | Relative BA study | Liver transplant (n=15); 0.4–10.6 years; two daily dose | Oral suspension of tacrolimus (OST) | Tacrolimus capsules (TC) | Poor study design | The PK studies were performed in two different groups of patients, without using a crossover design or a matched contemporaneous control group. Comparison of PK data showed a lower oral absorption of OST when compared with TC. |
| 80 | Stavudine Lamivudine Nevirapine | Vanprapar 2010  (67) | Relative BA study; Phase I/II, 2 stage, 2 arm, randomized, open-label | HIV-1 infected Thai children (n=34); ≥6 months to <13 years of age | Chewable tablets | Oral solutions | Poor study design | In view of the known variability of Nevirapine exposure in children, allowing the study to continue into stage II, where exposure boundaries were more stringent but the sample size was larger. The chewable tablets was safe and provided therapeutically adequate plasma drug exposures in human immunodeficiency virus-infected children. Substituting the chewable tablets for liquid formulations can simplify antiretroviral therapy. |
| 81 | Lopinavir (LPV) Ritonavir ® | Musiime 2014  (95) | Relative BA study; Open, randomized, phase I, 2-period crossover relative bioavailability trial | HIV infected infants and Ugandan children;3 to < 12 months (cohort A); children aged 1–4 years (cohort B); children aged 4 to ,13 years (cohort C);infants/children were included in cohort A (n = 19)/B (n = 26)/C (n = 32) | Minitab Sprinkles | Syrup; tablets | Poor study design including small sample size | PK parameters was moderate to high with all formulations  Apart from relatively small numbers (59 children with 2 pharmacokinetic profiles), 1 limitation is that our study included only Ugandan children |
| 82 | Efavirenz | ter Heine 2008  (43) | Relative BA study | HIV-1 infected children (n =33; females =16); median age of 33 children- 6.5 years (0.9-19 years) | Efavirenz Liquid formulation (30 mg/ml) | Capsules/tablets (200, 250, 300, 350, 400 & 600 mg) | Poor study design and small sample size | Our study is limited by the number of children included. Non-compliance could not be ruled out and could have influenced the results. |
| 83 | Tacrolimus | Jacobo-Cabral 2014  (35) | BE study | Renal transplant recipients; Prograf group (n=29); Limustin (n=9);Prograf group- 12-16 years; Limustin-14.5-17 years; Oral twice a day; Fasted | Limustin | Prograf | Poor study design | Parallel study design also-Our design, however, also exhibits limitations that should be noted. A parallel group design was used. A crossover design is more appropriate for bioavailability comparisons between formulations. However, we chose not to use a crossover design as immunosuppressant formulation switching is not recommended during treatment of organ transplant recipients and pediatric patients are considered as a high-risk population. Hence, tacrolimus formulations were not assigned by randomization. |

Acronyms used- AUC- Area under the curve; BA- bioavailability; CyA- Cyclosporine A; CYA- oil-based predecessor of CsA; C2h- Concentration at 2 hours time; CBZ- Carbamazepine; Cmax- Maximum plasma concentrations; BE-Bioequivalence; FDC- Fixed dose combination tablets; HIV- human immune deficiency virus; LT-4- Levothyroxine; MEC- microemulsified galenic formulation of CsA; 6-MP- 6 mercaptopurine; NVP-Nevirapine; NEO- Neoral; OLT- Orthotopic liver transplantation; PK- Pharmacokinetics; PHT-Phenytoin; RMP- Rifampicin; SR- sustained release; SIM- Sandimmune
